# Preliminary investigation of a significant national *Cryptosporidium* exceedance in the United Kingdom, August 2023 and ongoing

**DOI:** 10.2807/1560-7917.ES.2023.28.43.2300538

**Published:** 2023-10-26

**Authors:** Lewis Peake, Thomas Inns, Christopher Jarvis, Grace King, Hussein Rabie, Joan Henderson, Adrian Wensley, Reece Jarratt, Christopher Roberts, Christopher Williams, Oghogho Orife, Lynda Browning, Matthew Neilson, Caitlin McCarthy, Paul Millar, Nicola Love, Kristin Elwin, Guy Robinson, Trish Mannes, Nick Young, Rachel Chalmers, Richard Elson, Roberto Vivancos

**Affiliations:** 1 United Kingdom Health Security Agency, London, the United Kingdom; 2 Public Health Wales, Cardiff, the United Kingdom; 3 Public Health Scotland, Edinburgh, the United Kingdom; 4 Health and Safety Committee (HSC) Public Health Agency, Belfast, Northern Ireland, the United Kingdom; 5 *Cryptosporidium* Reference Unit, Public Health Wales, Swansea, the United Kingdom; 6 National Institute for Health and Care Research (NIHR) Health Protection Research Unit in Gastrointestinal Infections, Liverpool, the United Kingdom; 7 NIHR Health Protection Research Unit in Emergency Preparedness and Response, London, the United Kingdom; 8 NIHR Health Protection Research Unit in Emerging and Zoonotic Infections, Liverpool, the United Kingdom

**Keywords:** Cryptosporidium, cryptosporidiosis, United Kingdom, swimming pools, travel

## Abstract

Routine laboratory surveillance has identified an unprecedented and ongoing exceedance of *Cryptosporidium* spp. across the United Kingdom, notably driven by *C. hominis* transmission, since 14 August 2023. Information from 477 reported cases in England and Wales, followed up with a standardised exposure questionnaire as of 25 September 2023, identified foreign travel in 250 (54%) of 463 respondents and swimming in 234 (66%) of 353 cases. A significant, common exposure has not yet been identified in first analyses.

The majority of disease caused by infection with *Cryptosporidium* in the United Kingdom (UK) is due to two species, *Cryptosporidium hominis* and *Cryptosporidium parvum*. National and international outbreaks can occur, but most outbreaks are localised and associated with private or public water supplies, swimming pools, animal contact, person-to-person spread or food consumption [1]. Here we describe the initial investigation into an unprecedented and ongoing nation-wide increase in cases of cryptosporidiosis, first noted in August 2023, and present findings from early descriptive analyses.

## Exceedance detection and enhanced surveillance

Across the UK, routine surveillance of cryptosporidiosis relies on mandatory laboratory notification. Since the International Organization for Standardization (ISO) week 33 2023 (starting on 14 August), and as at week 39 2023 (ending on 1 October), the combined weekly number of laboratory notifications of *Cryptosporidium* spp. detections in England, Wales and Northern Ireland has exceeded the expected upper threshold (Figure); an exceedance in Scotland has only been present since ISO week 39.

**Figure:**
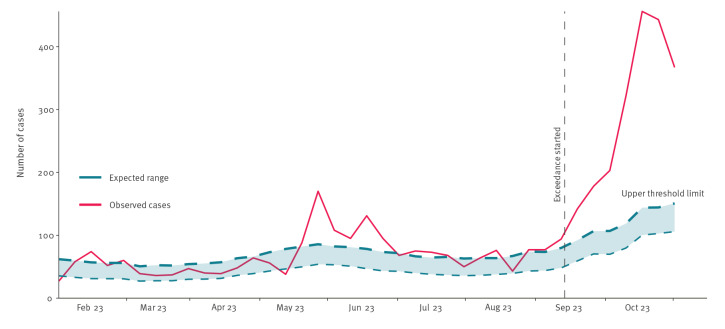
Laboratory notifications of *Cryptosporidium* species in England, Wales and Northern Ireland, by week of specimen, 2023

Observed case numbers are compared to an exceedance threshold as derived from the Farrington Flexible algorithm, using published methodology [10]; the model can account for seasonal effects and changing trends. The upper threshold limit reflects the 99.5% percentile of a negative binomial distribution for the expected case frequency.

Given the scale and geographical spread of the exceedance across regions and nations of the UK, a single local exposure is an unlikely cause.

In England, local practice varies in the routine collection of exposure information in cryptosporidiosis cases. To allow for consistent capture of information as part of this investigation, a standardised electronic questionnaire was deployed. This includes questions on foreign travel, food and water exposures and interaction with animals. Data collection through this method has been ongoing since ISO week 38 (and in retrospect), for cases meeting the following definition: any person resident in England, with a clinical specimen dated 14 August 2023 or later, positive for *Cryptosporidium* (any species). In Wales, a standard questionnaire for gastrointestinal disease has been in place since before the exceedance.

## Reference laboratory genotyping

Across England and Wales, standard practice is that *Cryptosporidium* spp. positive stool samples from local laboratories are referred to the *Cryptosporidium* Reference Unit (CRU, Swansea, UK) for genotyping. In 2022 [2], ca 70% of specimens were referred (2,913 in total, of which 2,698 were successfully genotyped); 63% (n = 1,694) were *C. parvum* and 34% (n = 919) *C. hominis*. Among genotyped specimens, 16% (n = 440) reported international travel, 65% (n = 284) of which were *C. hominis*.

Subtyping beyond species level is done to investigate clusters or outbreaks by sequencing the *gp60* gene [3] using real-time PCR amplicons; this is underway as part of this investigation for cases who have completed a questionnaire.

## Descriptive analysis of laboratory-reported cases

Between week 33 and 39, there have been 2,411 laboratory confirmed cases of cryptosporidiosis in the UK (2,032 in England, 163 in Wales, 127 in Scotland and 89 in Northern Ireland). Cases have been seen across almost all regions of the four UK nations.

Reference laboratory data suggest the exceedance is particularly due to *C. hominis* transmission (Table 1). Investigation by *gp60* sequencing of an initial 76 specimens from *C. hominis* cases shows that five are family Ia, 21 are family Ib, 16 are family Id, six are Ie and 28 are If. Of the most prevalent *gp60* families, only a single If subtype was found, IfA12G1R5, whereas there were three Ib subtypes, IbA9G3, IbA10G2 and IbA13G3, and three Id subtypes, IdA14, IdA16 and IdA17G1. The high prevalence of IfA12G1R5 is unusual in the UK [1,4].

**Table 1:** Notified cases of cryptosporidiosis, United Kingdom, ISO weeks 33–39 2023^a^ (n = 2,411)

Characteristics	England	Wales	Scotland	Northern Ireland
Local laboratory				
All *Cryptosporidium* species	2,032	163	127	89
Exceedance threshold first reached	Week 33	Week 35	Week 39	NA
Reference laboratory^b^				
*C. parvum*	431		10	0
*C. hominis*	929		25	0
Other *Cryptosporidium* species	19		0	0
Not typable	32		0	0
Not yet processed	784		92	89

ISO: International Organization for Standardization; NA: not available.

^a^ At time of publication and subject to change.

^b^ In England and Wales, local laboratories are requested to refer all *Cryptosporidium* positive stool samples for genotyping to the same reference laboratory. Scotland has a separate reference laboratory, and Northern Ireland specimens are not routinely genotyped. There is a lag between initial laboratory notification and genotyping data. Where specimen date is not available, date received by the reference laboratory is used.

## Descriptive analysis of questionnaire data

In England, as of 2 October, 406 standardised exposure questionnaires have been completed for individuals reporting a symptom onset date of 1 August (ISO week 31) or later, with species data available for 235 (58%). Comparable questionnaire data from Wales are available for an additional 71 cases reporting symptom onset from 14 August (ISO Week 33).

Foreign travel was reported by 250 (54%) of 463 *Cryptosporidium* spp. cases and by 139 (65%) of 213 *C. hominis* cases (Table 2). Of the 394 cryptosporidiosis cases resident in England who provided information on travel, 215 (55%) reported foreign travel in the 14 days preceding their illness, of which 96 (45%) noted travel to Spain (Spanish mainland and/or the Balearic Islands).

**Table 2:** Characteristics and exposures of cryptosporidiosis cases in weeks 31–39 in England and weeks 33-39 in Wales, United Kingdom, 2022 (n = 209) and 2023 (n = 477)

Characteristics	*Cryptosporidium* spp.				*C. hominis* W31–39 2023^b^ n = 215		*C. parvum* W31–39 2023^b^ n = 85	
	W31–39 2022^a^ n = 209		W31–39 2023^b^ n = 477					
	n	%	n	%	n	%	n	%
Age (years)	n = 208		n = 475		n = 215		n = 85	
0–4	35	17	68	14	34	16	11	13
5–9	46	22	91	19	44	20	13	15
10–19	30	14	77	16	36	17	16	19
20–39	61	29	141	30	60	28	26	31
40–59	26	13	67	14	36	17	7	8
60–79	8	4	30	6	5	2	12	14
> 80	2	1	1	< 1	0	0	0	0
Sex	n = 209		n = 476		n = 215		n = 85	
Male	88	42	218	46	101	47	36	42
Female	121	58	258	54	114	53	49	58
Symptom duration (days)	n = 95		n = 224		n = 91		n = 46	
0–4	6	6	26	12	11	12	6	
5–9	33	35	73	33	30	33	14	
10–14	42	44	85	38	31	34	21	
15–19	12	13	21	9	11	12	3	
≥ 20	2	2	19	8	8	9	2	
Foreign travel^c^	n = 208		n = 463		n = 213		n = 85	
Yes	118	57	250	54	139	65	24	28
No	90	43	213	46	74	35	61	72
Swimming^c^	n = 111		n = 353		n = 155		n = 70	
Yes	61	55	234	66	124	80	26	37
No	50	45	119	34	31	20	44	63
Farm animal exposure^c^	n = 110		n = 384		n = 164		n = 73	
Yes	20	18	71	18	34	21	14	19
No	90	82	313	82	130	79	59	81
Food exposures^c^								
Salad at home			n = 164		n = 49		n = 28	
Yes	NA		77	47	17		19	
No			87	53	32		9	
Pasteurised cow’s milk			n = 182		n = 57		n = 27	
Yes	NA		120	66	37		21	
No			62	34	20		6	
Tap water			n = 219		n = 71		n = 37	
Yes	NA		163	74	53	75	30	
No			56	26	18	25	7	
Bottled water			n = 231		n = 77		n = 41	
Yes	NA		137	59	55	71	17	
No			94	41	22	29	24	
Ate at a restaurant			n = 346		n = 136		n = 58	
Yes	NA		210	61	89	65	36	
No			136	39	47	35	22	

*C*.: *Cryptosporidium*; NA: not available; spp.: multiple species; W: ISO week.

^a^ Cases from **four** regions in England and Wales where questionnaire data were available and symptom onset was between ISO week 31 and 39 2022. Data may be incomplete, especially for ISO weeks 31 and 32. Heterogeneity in questionnaires used in 2022 means that comparisons to 2023 data should be done with caution.

^b^ Cases from **all** regions in England (between ISO week 31 and 39 2023) and Wales (between ISO week 33 and 39 2023), where questionnaire and genotyping data were available, based on symptom onset. Percentages not included where n < 60.

^c^ In 14 days before onset of illness.

Comparisons to available questionnaire data from four regions in England and Wales from a similar period in 2022 (Table 2), which is done cautiously given the heterogeneity in surveys used in that year and the availability of data, suggest the 2023 exceedance may be associated with greater exposure to swimming in the 14 days preceding illness (odds ratio (OR) = 1.61; 95% confidence interval (CI): 1.04–2.48), despite minimal difference in the proportion reporting foreign travel.

Analysis of 2023 questionnaires for potential setting exposures (e.g. swimming pools or water parks) has not detected significant case clusters that could alone explain the large exceedance. However, to date, two local exposures mutual to a small number of cases have been identified and have been actively investigated by environmental health teams in local authorities.

## Public health measures

Routine public health measures to prevent transmission of common gastrointestinal infections are in place. As yet, survey responses have not identified common specific exposures or settings that may explain large numbers of cases. Public health action within the UK has included stakeholder communications to raise awareness and highlight the importance of avoiding use of swimming pools while symptomatic, and in the 14 days after resolution of symptoms. We are working with the National Travel Health Network and Centre (NaTHNaC) to ensure appropriate travel advice is available. We are liaising with international partners, including European Centre for Disease Prevention and Control (ECDC), to inform further investigations.

Identification and investigation of any local common exposures will be ongoing, within the remit of local health protection teams. Data collection, and analysis at a national level, will continue until the exceedance resolves.

## Discussion

We have described an ongoing and unusual national exceedance in disease caused by *Cryptosporidium* spp., most notably driven by *C. hominis* incidence, for which no single exposure has been identified. While *C. hominis* infections (dominantly transmitted person-to-person via the faecal-oral route) are expected to increase at this time of year, the magnitude of the increase warrants further investigation.

Responses to surveillance questionnaires suggest many cases may relate to international travel, most notably to Spain and other Mediterranean countries. The exceedance may in part reflect increasing summer travel to the European continent in 2023, although travel trend data are not yet available for this year (historical data suggests Spain accounted for 40–50% of UK foreign travel in the years before the COVID-19 pandemic, and 33% of travel in 2022 [5]). Investigations in previous similar exceedances found international travel to be a common exposure [6].

Our initial findings would suggest that swimming (either in the UK or abroad), including the use of pools, and foreign travel to a variety of destinations may underlie the current increase. However, at this stage other sources, for example contaminated food, cannot be excluded as contributing to the exceedance. Ongoing *gp60* subtyping may yield large unexplained clusters warranting further investigation. Furthermore, continuation of a sentinel scheme to detect genetic clusters of *C. parvum* by multilocus variable number tandem repeat analysis (MLVA) [7] initiated as a pilot in Wales and the north-west of England [8] has yet to identify any notable clusters during this exceedance.

This exceedance has reinforced the considerable disease burden that can result from cryptosporidiosis (56% of the 224 cases responding to the questionnaire and reporting an illness duration of more than 10 days), as well as the typical age-sex distribution of *Cryptosporidium* spp. infections. Previous studies have shown that *Cryptosporidium* spp. are highly transmissible within households, particularly those with children, and that a notable number of cases are under-reported [9]. The importance and utility of standard surveillance approaches within countries has also been demonstrated; rapid roll out of a single questionnaire has allowed for hypothesis generation and analysis at a national level.

## References

[1] Chalmers RM Robinson G Elwin K Elson R . Analysis of the Cryptosporidium spp. and gp60 subtypes linked to human outbreaks of cryptosporidiosis in England and Wales, 2009 to 2017. Parasit Vectors. 2019;12(1):95. 10.1186/s13071-019-3354-6 30867023 PMC6417012

[2] Public Health Wales Microbiology. Annual report of referrals and Cryptosporidium genotyping; England and Wales, 2022. Swansea: Public Health Wales; 2023. Available from: https://phw.nhs.wales/services-and-teams/cryptosporidium-reference-unit/annual-report-of-referrals-and-cryptosporidium-genotyping-england-and-wales-2022

[3] Strong WB Gut J Nelson RG . Cloning and sequence analysis of a highly polymorphic Cryptosporidium parvum gene encoding a 60-kilodalton glycoprotein and characterization of its 15- and 45-kilodalton zoite surface antigen products. Infect Immun. 2000;68(7):4117-34. 10.1128/IAI.68.7.4117-4134.2000 10858229 PMC101708

[4] Bacchetti R Connelly L Browning L Alexander CL . Changing molecular profiles of human cryptosporidiosis cases in Scotland as a result of the coronavirus disease, COVID-19 pandemic. Br J Biomed Sci. 2023;80:11462. 10.3389/bjbs.2023.11462 37701073 PMC10493326

[5] Office for National Statistics (ONS). Travel trends: 2022. Newport: ONS; 26 May 2023. Available from: https://www.ons.gov.uk/peoplepopulationandcommunity/leisureandtourism/articles/traveltrends/2022

[6] Fournet N Deege MP Urbanus AT Nichols G Rosner BM Chalmers RM Simultaneous increase of Cryptosporidium infections in the Netherlands, the United Kingdom and Germany in late summer season, 2012. Euro Surveill. 2013;18(2):20348. 10.2807/ese.18.02.20348-en 23324424

[7] Robinson G Pérez-Cordón G Hamilton C Katzer F Connelly L Alexander CL Validation of a multilocus genotyping scheme for subtyping *Cryptosporidium parvum* for epidemiological purposes. Food Waterborne Parasitol. 2022;27:e00151. 10.1016/j.fawpar.2022.e00151 35498551 PMC9043402

[8] Risby Robinson G Chandra N King G Vivancos R Smith R Application of a new multilocus variable number tandem repeat analysis (MLVA) scheme for the seasonal investigation of Cryptosporidium parvum cases in Wales and the north west of England, spring 2022. Curr Res Parasitol Vector Borne Dis. 2023;100151. 10.1016/j.crpvbd.2023.100151 PMC10665698 38021189

[9] McKerr C Chalmers RM Elwin K Ayres H Vivancos R O’Brien SJ Cross-sectional household transmission study of Cryptosporidium shows that C. hominis infections are a key risk factor for spread. BMC Infect Dis. 2022;22(1):114. 10.1186/s12879-022-07086-y 35105330 PMC8807379

[10] Noufaily A Enki DG Farrington P Garthwaite P Andrews N Charlett A . An improved algorithm for outbreak detection in multiple surveillance systems. Stat Med. 2013;32(7):1206-22. 10.1002/sim.5595 22941770

